# Quantum Coherence and Total Phase in Semiconductor Microcavities for Multi-Photon Excitation

**DOI:** 10.3390/nano12152671

**Published:** 2022-08-03

**Authors:** Abeer S. Altowyan, Kamal Berrada, Sayed Abdel-Khalek, Hichem Eleuch

**Affiliations:** 1 Department of Physics, College of Science, Princess Nourah bint Abdulrahman University, P.O. Box 84428, Riyadh 11671, Saudi Arabia; asaltowyan@pnu.edu.sa; 2 Department of Physics, College of Science, Imam Mohammad Ibn Saud Islamic University (IMSIU), P.O. Box 5701, Riyadh 11432, Saudi Arabia; 3 The Abdus Salam International Centre for Theoretical Physics, Strada Costiera 11, 34151 Miramare-Trieste, Italy; 4 Department of Mathematics and Statistics, College of Science, Taif University, P.O. Box 11099, Taif 21944, Saudi Arabia; sayedquantum@yahoo.co.uk; 5 Department of Mathematics, Faculty of Science, Sohag University, Sohag 82524, Egypt; 6 Department of Applied Physics and Astronomy, University of Sharjah, Sharjah 27272, United Arab Emirates; hichemeleuch@yahoo.fr; 7 Department of Applied Sciences and Mathematics, College of Arts and Sciences, Abu Dhabi University, Abu Dhabi 59911, United Arab Emirates; 8 Institute for Quantum Science and Engineering, Texas A&M University, College Station, TX 77843, USA

**Keywords:** semiconductors, four-photon excitation, microcavities, quantum coherence, fidelity, total phase

## Abstract

We examine how the weak excitation regime of a quantum well confined in a semiconductor microcavity (SM) influences the dynamics of quantum coherence and the total phase. We analyze the impact of the physical parameters on different quantumness measures, and illustrate their numerical results. We show that the amount of the coherence and total phase in the SMs for multi-photon excitation can be improved and controlled by the strength of the field, exciton-photon coupling, cavity dissipation rate, and excitonic spontaneous emission rate. We illustrate how the fidelity varies depending on the physical parameters. These results might have far-reaching ramifications not just in quantum information processing and optics, but also in physics at large.

## 1. Introduction

Coherence is not only the main resource in different areas of quantum information and optics, but also be appointed a fundamental feature of quantum physics [1,2,3,4]. The quantum coherence, which is characterized by the off-diagonal elements of the system density matrix, is related to the concept of the superposition principle. Recently, several studies on the quantification of coherence received considerable attention [5,6,7,8,9]. Because each system unavoidably interacts with its external environs, the quantity of coherence is highly crisp and sensitive to environmental exposure, similar to quantum correlations. This indicates that it is not easy to generate, maintain, and manipulate the coherence in quantum systems. Thus, it is needful and extremely important to generate and maintain quantum coherence. To exploit coherence, we need to measure the amount of coherence for quantum states. Fortunately, Baumgratz et al. [10] have proposed a suitable context for the theory of quantum coherence considering the definition of incoherent operations, incoherent states, and arbitrary valid coherence measures. A variety of research has been conducted in this field, with some focusing particularly on the features of certain coherence measures [11,12,13,14,15,16,17] and more recently by exploiting the quantum uncertainty relation by using quantum coherence [18,19,20,21].

Recently, considerable studies have concentrated on the quantum phases. These studies include the Pancharatnam phase [22] in the study of the interference effect of light waves and the geometric phase (GP) introduced by Berry [23]. This is a property of quantum mechanics that is determined by the path considered in space, which includes all potential quantum states of the system. For partial cycles, the definition of phase change was proposed by Jordan [24]. The idea of Pancharatnam was utilized by Samuel and Bhandari [25,26], to prove that the presence of Berry’s phase does not need the system to be cyclic or unitary [27,28], and that quantum measurements can interrupt it. Actually, quantum computation models founded on the density operator as a quantum state have been established [29]. The GP shift has been shown to be useful for the production of fault-tolerance phase shift gates [30]. The basic definition has been subjected to several generalizations [31]. The underlying theory of GPs as well as their significance in quantum mechanics theory [32,33] has been explored. Several experiments with a Michelson interferometer have been suggested for observing the nonlinearity of the Pancharatnam phase [34].

It is crucial to analyze the interaction characteristics of a quantum system in order to understand how its behavior changes, especially when examining the aspects of light–matter interaction. The study of this bipartite system began when scientists discovered the spectral lines in the solar spectrum qualify as light–matter interaction studies. Currently, light–matter interaction is well understood, and its theory was established to develop outgrowth lasers, quantum optics, and quantum computing, and other topics in physics. In the last decades, several works have examined the light–matter interaction in the presence of the dissipation effect, which should be contemplated in the experimental implementation [35,36]. The dissipation is treated in the presence of an external environment in the framework of energy dissipation, wherein the system loses energy during evolution.

The formation of polaritons in the SM arises from the coupling of photons and excitons, whose properties are determined by the mix of light and matter [37,38,39,40,41,42,43,44,45]. The optical fields are confined by the microcavity, resulting in non-classical phenomena. In SMs, squeezing, bistability, chaos, superfluidity, and entanglement have all been predicted, with some of these phenomena being observed. Experimental evidence for bistability in the existence of a strong-coupling regime has recently been considered [46], wherein nonlinear patterns appearing on the transverse plane are analyzed, and the significant characteristics of the empirical outcomes can be understood by investigating the interaction between polaritons. More recently, an experimental and theoretical study of polariton condensates in a planar semiconductor microcavity was considered, wherein the polaritons are characterized as hybrid light–matter quasiparticles obtained by considering the strong coupling between quantum-well excitons and cavity photons [47]. Specifically, the authors measure the coherence of the superposition of Fock states and provide the quantum-coherent interfacing capabilities of two platforms, emitted light and polariton system, thus addressing the problem of interconnecting devices in a way that is advantageous for quantum information science and technology. The concept of quantum coherence from the theory of quantum information is different from other nonclassical quantum quantifiers that have formerly been considered in the systems of condensed-matter physics. In the present manuscript, we examine how in the weak excitation regime (WER) a quantum well contained in an SM influences quantum coherence dynamics and the total phase (TP). We will analyze the effect of the parameters of the system model on the different measures of quantumness, and illustrate their numerical results. We will discuss how the quantity of coherence and TP in SMs for multi-photon excitation (MPE) can be increased and adjusted based on system parameters such as the field, cavity dissipation rate, excitonic spontaneous emission (ESE) rate, and photon-exciton coupling. We also illustrate how fidelity varies with various physical parameter values.

The following is the structure of the current manuscript. The physical model as well as the physical system’s evolution equations is presented in Section 2. Section 3 exhibits the measures of quantumness considered in this manuscript. In Section 4, we display and discuss the main results. The main conclusions are presented in the last section.

## 2. Physical Model

Here, we consider a quantum well inside an SM in the context of WRE. The system Hamiltonian without spin effects has the form [48,49,50,51,52,53,54]

(1) $$H=\hbar \omega_{e}b^{†}b+\hbar \omega_{p}a^{†}a+ı\hbar g^{\prime }\left(a^{†}b-b^{†}a\right)+\hbar \alpha^{\prime }b^{†}b^{†}bb+ı\hbar \left(\varepsilon^{\prime }e^{ı\omega t}a^{†}-h.c\right)+H_{r},$$

where the operator a(b) represents the annihilation operator for the photonic (excitonic) fields and satisfy that $\left[a,a^{†}]=1;[b,b^{†}\right]=1$. Also, $\omega_{p}$ ($\omega_{e}$) designs the photonic (excitonic) frequencies modes of the cavity field.

In Equation (1), the first two terms represent the cavity photon and exciton energies whereas the third term refers the exciton–photon coupling constant $g^{\prime }$. The parameter $\alpha^{\prime }$ represents the strength of the non-linear excitonic interaction [55,56]. The last term describes the external driving where $\varepsilon^{\prime }$ and ω design respectively the amplitude and frequency of the coherent field. In this work, we focus on the resonant condition $\omega =\omega_{p}=\omega_{e}$ without nonlinear dissipations [57], and the master equation is expressed as [58,59,60,61,62]

(2) $$\frac{\partial \rho }{\partial t}+i\alpha \left(b^{†}b^{†}bb\rho -\rho b^{†}b^{†}bb\right)=L\rho +g\left[\left(a^{†}b-b^{†}a\right),\rho \right]+\varepsilon \left(a^{†}-a\right),\rho ].\;$$

Here, all constant parameters are normalized to $1/\tau_{c}$ as: $g=g^{\prime }\tau_{c},\varepsilon =\varepsilon^{\prime }\tau_{c},\alpha =\alpha^{\prime }\tau_{c}$. Lρ designs the dissipation term linked to $H_{r}$ describing the dissipation due to the ESE rate γ/2 and to the cavity dissipation rate κ:

(3) $$L\rho =-\gamma /2\left(b^{†}b\rho +\rho b^{†}b-2b\rho b^{†}\right)-\kappa \left(a^{†}a\rho +\rho a^{†}a-2a\rho a^{†}\right).$$

We consider two approximations. The first approximation is based on neglecting the terms $2a\rho a^{†}$ and $2b\rho b^{†}$ in Equation (3). This could be justified by the fact that in the WER the purity of the state is maintained when the excitation is so low that, over a period of a few correlation times, the quantum state simply evolves and the probabilities of spontaneous emission and cavity loss are minimal. We may disregard the terms $2a\rho a^{†}$ and $2b\rho b^{†}$ in this case because they contribute to the density matrix’s mixed-state nature [63,64]. The density matrix can then be factorized as a pure state. This approximation is formally evidenced by extending the density operator components and the related equations of motion in powers of to a dominating order of ε/κ [63,64].In this case, we get

(4) $$\frac{d|u\left(t\right)\rangle }{dt}=-\frac{i}{\hbar }H_{eff}|u\left(t\right),$$

where $H_{eff}$ is the effective non-Hermitian Hamiltonian [63,64,65]

(5) $$H_{eff}=ı\hbar g\left(a^{†}b-b^{†}a\right)+\hbar \alpha b^{†}b^{†}bb+ı\hbar \varepsilon \left(a^{†}-a\right)-ı\hbar \kappa a^{†}a-ı\hbar \frac{\gamma }{2}b^{†}b.$$

The second approximation is to limit the number of excitations inside the cavity. In the WER, we can write the wave function |u(t)〉 as a superposition of product of excitonic and photonic states and retain up to four states, which can be justified by the excitation of the cavity [56,57,58,59,60,61,62,63,64,65]

(6) $$\begin{array}{c} \left|u\left(t\right)\rangle =\right|0\rangle ⊗(R_{00}\left|0\rangle +R_{01}\right|1\rangle +R_{02}\left|2\rangle +R_{03}\right|3\rangle +R_{04}|4\rangle )\\ +|1\rangle ⊗\left(R_{10}\left|0\rangle +R_{11}\right|1\rangle +R_{12}\left|2\rangle +R_{13}\right|3\rangle \right)\\ +|2\rangle ⊗(R_{20}\left|0\rangle +R_{21}\right|1\rangle +R_{22}|2\rangle )\\ +\left|3\rangle ⊗\left(R_{30}\left|0\rangle +R_{31}\right|1\rangle \right)+\right|4\rangle ⊗R_{40}|0\rangle . \end{array}$$

The coefficients $R_{ij}$ are obtained by solving the following system of ODE:

(7) $$\begin{array}{l} \frac{dR_{00}}{dt}=-\varepsilon R_{10}\\ \frac{dR_{01}}{dt}=-\frac{1}{2}\gamma R_{01}-\varepsilon R_{11}-gR_{10}\\ \frac{dR_{10}}{dt}=gR_{01}-kR_{10}+\varepsilon \left(R_{00}-\sqrt{2}R_{20}\right)\\ \frac{dR_{11}}{dt}=-\left(\kappa +\frac{\gamma }{2}\right)R_{11}+\sqrt{2}g\left(R_{02}-R_{20}\right)+\varepsilon \left(R_{01}+\sqrt{2}R_{21}\right)\\ \frac{dR_{20}}{dt}=\varepsilon \left(\sqrt{2}R_{10}-\sqrt{3}R_{30}\right)+\sqrt{2}gR_{11}-2\kappa R_{20}\\ \frac{dR_{02}}{dt}=-\gamma R_{02}-\varepsilon R_{12}-\sqrt{2}\left(gR_{11}+\sqrt{2}i\alpha R_{02}\right)\\ \frac{dA_{03}}{dt}=-\frac{3}{2}\gamma R_{03}-\varepsilon R_{13}-\sqrt{3}\left(gR_{12}+2\sqrt{3}i\alpha \right)R_{03}\\ \frac{dR_{30}}{dt}=\sqrt{3}\left(gR_{21}-\sqrt{3}\kappa R_{30}\right)+\varepsilon \left(\sqrt{3}R_{20}-2\varepsilon R_{40}\right)\\ \frac{dR_{12}}{dt}=-\left(\gamma +\kappa +2i\alpha \right)R_{12}+\varepsilon \left(R_{02}-\sqrt{2}R_{22}\right)+g\left(\sqrt{3}R_{03}-2R_{21}\right)\\ \frac{dR_{21}}{dt}=-\left(\frac{\gamma }{2}+2\kappa \right)R_{21}+\varepsilon \left(\sqrt{2}R_{11}-\sqrt{3}R_{31}\right)+g\left(2R_{12}-\sqrt{3}R_{30}\right)\\ \frac{dR_{04}}{dt}=-2\left(\gamma R_{04}+6i\alpha R_{04}+gR_{13}\right)\\ \frac{dR_{40}}{dt}=2\left(\varepsilon R_{30}+gR_{31}-2\kappa R_{40}\right)\\ \frac{dR_{22}}{dt}=2\left(\frac{1}{2}\gamma +\kappa +i\alpha \right)R_{22}+\sqrt{6}g\left(R_{13}-R_{31}\right)+\varepsilon \sqrt{2}R_{12}\\ \frac{dR_{13}}{dt}=-3\left(\frac{1}{2}\gamma +\frac{1}{3}\kappa +2i\alpha \right)R_{22}+\varepsilon R_{03}+2g\left(R_{04}-\sqrt{\frac{3}{2}}R_{22}\right)\\ \frac{dR_{31}}{dt}=-3\left(\kappa +\frac{1}{6}\gamma \right)R_{22}+\varepsilon \sqrt{3}R_{21}+g\left(\sqrt{6}R_{22}-2R_{40}\right)\; \end{array}$$

We consider that the initial wavefunction is defined as the vacuum state $R_{00}\left(t=0\right)=1$ and for i≠0 and j≠0,

(8) $$R_{ij}\left(t=0\right)=0.$$

For pure state, $\rho_{ph,exc}=\left|u\left(t\right)\rangle \langle u\left(t\right)\right|$ and the reduced density operator of the photons and exciton system is

(9) $$\rho_{ph}=tr_{exc}\left(\left|u\left(t\right)\rangle \langle u\left(t\right)\right|\right),\rho_{exc}=tr_{ph}\left(\left|u\left(t\right)\rangle \langle u\left(t\right)\right|\right).$$

In the next section, the obtained equation will be used for determining the quantum coherence and the total phase.

## 3. Coherence, Fidelity and Total Phase

In this research, we use a specific measure to investigate the phenomena of coherence in SMs for MPE based on the $l_{1}$ norm. The $l_{1}$ absluate value of coherence $C_{l_{1}}$ is defined as [10]

(10) $$C_{l_{1}}\left(\rho \right)=\underset{\chi \in I}{\min}D\left(\rho ,\chi \right),$$

where $D\left(\rho ,\chi \right)=|\left|\rho -\chi \right|{|}_{1}$ represents the distance from the quantum state ρ and a set of incoherent states I. We can check that the measure verifies the axiomatic definition of coherence measures suggested by Baumgratz et al. [10]. In the case of a d-dimensional system, it satisfies $0\leq C_{l_{1}}\leq d-1$.

In what follows, we will be interested by another important quantity in this paper, which is the fidelity. For a pure state, it is given by

(11) ξ(t)=Tr{ρ(0)ρ(t)},

which verifies the inequality 0≤ξ(t)≤1 and takes the maximal value when ρ(0)=ρ(t). The larger the fidelity is, the smaller distinguishable of the states are. These features made this quantity to be a measure of how well a state vector can be kept during the dynamics.

When a system evolves from an initial state to a final state, the evolution is noncyclic if the final state can’t be acquired by multiplying the initial state by a number. Consider the state |ψ(0)〉 evolves to a state |ψ(t)〉. If the inner product is

(12) $$F(t)=\langle \psi (0)|\exp[-i\int_{0}^{t}H(\tau )d\tau ]|\psi (0)\rangle ,$$

and by rewriting Equation (12) as F(t)=Γexp(iγ) where Γ is a real, thus the angle γ defines the noncyclic phase as a result of the evolution from state ket |ψ(0)〉 to the state ket |ψ(t)〉. During an arbitrary evolution, the total phase acquired of a wave function from the state ket |ψ(0)〉 to $\exp\left[-i\int_{0}^{t}H_{I}(\tau )(\tau )d\tau \right]|\psi (0)\rangle$ is given by [66,67,68,69]

(13) $$\phi_{G}=\arg\left[F\left(t\right)\right].$$

## 4. Numerical Results and Discussion

In the present section, we display the impact of the system parameters on the dynamical behaviour of the quantum coherence, fidelity, and TP in the SM for MPE.

**Figure 1:**
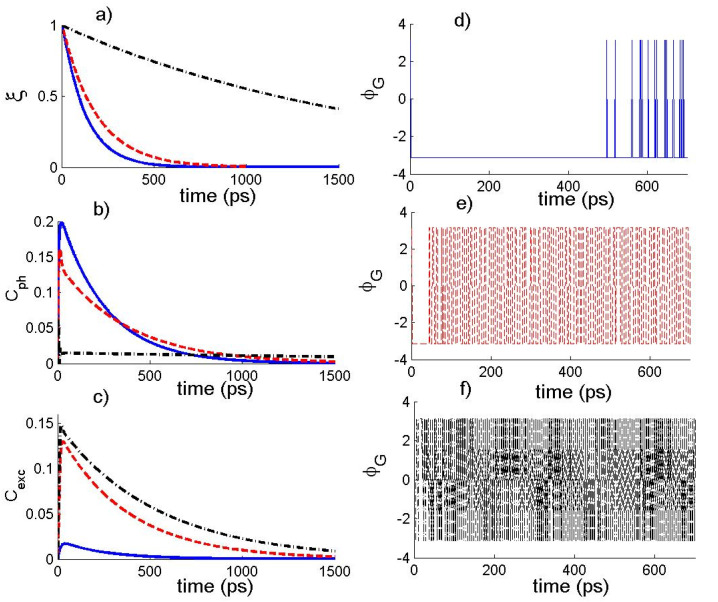
The temporal behavior of the (**a**) state fidelity ξ(t), (**b**) photons state coherence $C_{\mathrm{ph}}\left(t\right)$, (**c**) exciton-state coherence $C_{\mathrm{exc}}\left(t\right)$ and (**d**–**f**) the total phase $\phi_{G}$ of the photon–exciton state for $\alpha =10^{-8}$, (κ,γ)=(0.2,0), ε=0.01κ, where g=0.01 (solid curve), g=0.1 (dashed curve), and g=0.5 (dash-dotted curve).

In order to show the effects of the coupling constant between the photons and exciton, we display the dynamics of the quantum coherence, fidelity, and TP in the absence of ESE rate γ. In Figure 1, we plot the functions ξ, $C_{ph}$, $C_{exc}$ and $\phi_{G}$ versus time considering different values of g with κ=0.2 and ε=0.01κ. The solid curve is for g=0.01, the dashed curve is for g=0.1, and the dash-dotted curve is for g=0.5. For small values of g, we can observe from the numerical results that the function $C_{l_{1}}$ initially rapidly increases to its maximal value, and then decreases exponentially with the time. The maximal value, $C_{{\mathrm{ph}}_{m}}$ and $C_{{\mathrm{exc}}_{m}}$, of coherence highly depends on g. The smaller g is, the larger (smaller) $C_{{\mathrm{ph}}_{m}}$ ($C_{{\mathrm{exc}}_{m}}$). Moreover, the coupling constant g leads one to protect the coherence at large time. In that time interval, the coherence loss of the exciton and photon state decreases with the increase of the coupling constant g. When the parameter g becomes significantly large, we can observe that the function $C_{ph}$ increases to the maximal value and then reaches a steady value during the evolution, and the function $C_{exc}$ exhibits a similar behavior for very small values of g. On the other hand, the fidelity of the photon–exciton state exponentially decreases with time. The decay rate of the fidelity is largely dependent on  g. The larger g is, the smaller loss of the fidelity during the evolution. The time variation of the function $\phi_{G}$ shows the parameter g does not really influence the amplitude of the TP during the evolution. In this context, the function $\phi_{G}$ initially attains a constant negative value that depends on the values of the coupling constant g and then it takes negative and positive values displaying a periodic behavior with rapid oscillatory between −π and π.

**Figure 2:**
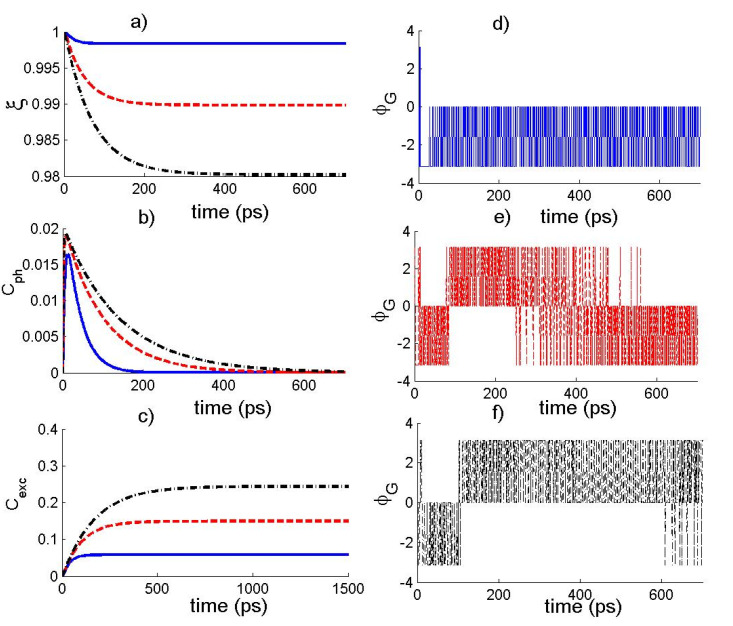
The temporal behavior of (**a**) state fidelity ξ(t), (**b**) photons-state coherence $C_{ph}\left(t\right)$, (**c**) exciton-state coherence $C_{\mathrm{exc}}\left(t\right)$ and (**d**–**f**) the total phase $\phi_{G}$ of the photon–exciton state for $\alpha =10^{-8}$, (g,γ)=(0.07,0), ε=0.01κ, where κ=0.2 (solid curve), κ=0.5 (dashed curve), and κ=0.8 (dash-dotted curve).

Let us now examine the influence of the parameter ε on the dynamics of the different quantifiers in the SM for MPE, we have depicted, in Figure 2, the dynamics of the different quantifiers as a function of time considering various values of ε in the absence of ESE rate γ. The solid curve is for κ=0.2, the dashed curve is for κ=0.5, and the dash-dotted curve is for κ=0.8. For each value of ε, we can see that the function $C_{ph}$ increases from the minimal value, reaches the maximal value, and then decreases with time, whereas the function $C_{exc}$ initially increases and then tends to attain a steady value. By increasing ε, the value of the functions $C_{ph}\;$ and $C_{exc}$ is amplified and then results in an enhancement of the photons-state and exciton-state coherence for MPE. On the other hand, we can note that the fidelity decreases as the time evolves and it attains a steady value for large time. Moreover, the decrease in the values of ε reduces the decay of the fidelity with the time. Concerning the behaviour of the TP, we can see from the figure that the function $\phi_{G}$ exhibits rapid oscillations between 0 and −π for small values of the parameter ε. The increase in the value of ε leads to the occurrence of oscillations of the function $\phi_{G}$ between the values −π and π, which shows that the amplitude of the coherent field may increase the TP amplitude and therefore may restrain the stabilization of the system during the time evolution. The obtained results indicate that the control and manipulation of the different measures of quantumness in semiconductor microcavities for MPE highly benefit from a considerable choice of the strength of the field and the photon–exciton coupling.

**Figure 3:**
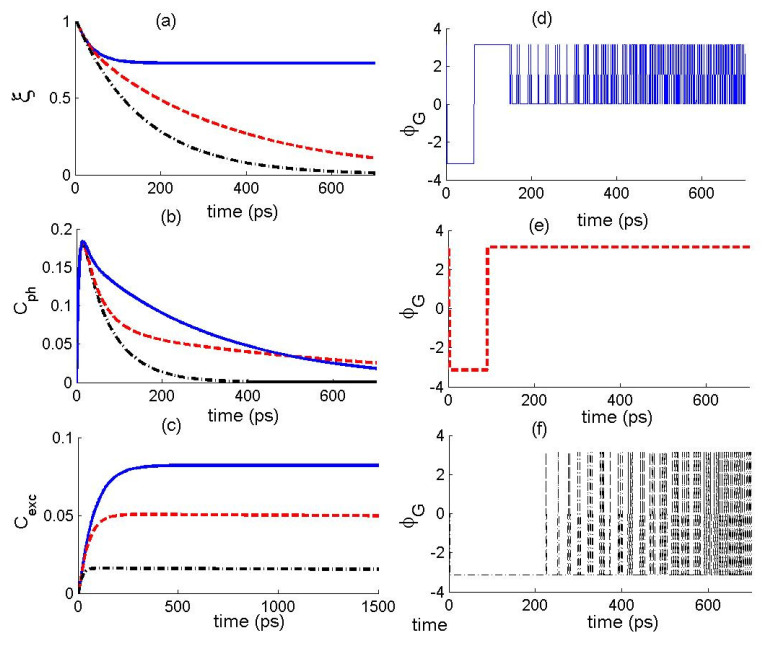
The temporal behavior of (**a**) state fidelity ξ(t), (**b**) photons-state coherence $C_{ph}\left(t\right)$, (**c**) exciton-state coherence $C_{\mathrm{exc}}\left(t\right)$ and (**d**–**f**) the total phase $\phi_{G}\;$ of the photon–exciton state for $\alpha =10^{-8}$, (g,κ)=(0.05,0.2), ε=0.01κ, where γ=0 (solid curve), γ=0.01 (dashed curve), and γ=0.1 (dash-dotted curve).

Finally, we display the impact of the ESE rate γ on the dynamics of the different quantifiers. In Figure 3, the quantum coherence, fidelity, and TP are plotted versus the time for different γ. The solid curve is for γ=0, dashed curve is for γ=0.01, and dash-dotted curve is for γ=0.1. From the figure, we can see that the quantifiers exhibit interesting features during the evolution, where their amount is largely dependent on the value of γ. The increase in the parameter γ can enhance the exciton-state coherence and suppress the photons-state coherence. On the other side, we can see that the fidelity decreases as the time evolves and that the increase in the values of γ reduces the decay of the fidelity with the time. Concerning the TP, we can see that the behavior of the function $\phi_{G}$ changes depending on the parameter γ.

## 5. Conclusions

In summary, we have examined how the WER of a quantum well confined in a SM influences the evolution of quantum coherence and the TP. We have investigated the effects of the physical parameters on the different measures of quantumness and illustrated their numerical results. We have shown that the amount of the coherence and TP in the SMs for MPE can be enhanced and controlled by a considerable selection of the strength of the field, cavity dissipation rate, rate of the ESE, and the coupling between the photons and exciton. Furthermore, we have considered the variation of the fidelity with respect to the parameters of the quantum system. We have shown how the appropriate selection of the field amplitude and the coupling between the exciton and photons in the existence of spontaneous emission greatly benefits the control and manipulation of different measures of quantumness in SMs for MPE. These results might have far-reaching ramifications not only for quantum information processing and optics, but also for the rest of the physics fields.

## Data Availability

Not applicable.
